# Do the Mechanical Properties of Calcium-Silicate-Based Cements Influence the Stress Distribution of Different Retrograde Cavity Preparations?

**DOI:** 10.3390/ma16083111

**Published:** 2023-04-14

**Authors:** Tarek Ashi, Raphaël Richert, Davide Mancino, Hamdi Jmal, Sleman Alkhouri, Frédéric Addiego, Naji Kharouf, Youssef Haïkel

**Affiliations:** 1 Department of Biomaterials and Bioengineering, INSERM UMR_S, Strasbourg University, 67000 Strasbourg, France; tarekachi@live.com (T.A.); mancino@unistra.fr (D.M.); 2 Hospices Civils de Lyon, PAM Odontologie, 69100 Lyon, France; richertg@gmail.com; 3 Laboratoire de Mécanique des Contacts et Structures, UMR 5259 CNRS/INSA Lyon, 69100 Lyon, France; 4 Department of Endodontics, Faculty of Dental Medicine, Strasbourg University, 67000 Strasbourg, France; 5 Pôle de Médecine et Chirurgie Bucco-Dentaire, Hôpital Civil, Hôpitaux Universitaire de Strasbourg, 67000 Strasbourg, France; 6 ICube Laboratory, Mechanics Department, UMR 7357 CNRS, University of Strasbourg, 67000 Strasbourg, France; jmal@unistra.fr; 7 Private Practice, 20097 Hamburg, Germany; slemanko@gmx.de; 8 Department Materials Research and Technology (MRT), Luxembourg Institute of Science and Technology (LIST), ZAE Robert Steichen, 5 Rue Bommel, L-4940 Hautcharage, Luxembourg; frederic.addiego@list.lu

**Keywords:** retrograde cavity, calcium silicate cement, stress distribution, compression strength

## Abstract

The aim of the present study was to investigate the influence of the mechanical properties of three different calcium-silicate-based cements on the stress distribution of three different retrograde cavity preparations. Biodentine™ “BD”, MTA Biorep “BR”, and Well-Root™ PT “WR” were used. The compression strengths of ten cylindrical samples of each material were tested. The porosity of each cement was investigated by using micro-computed X-ray tomography. Finite element analysis (FEA) was used to simulate three retrograde conical cavity preparations with an apical diameter of 1 mm (Tip I), 1.4 mm (Tip II), and 1.8 mm (Tip III) after an apical 3 mm resection. BR demonstrated the lowest compression strength values (17.6 ± 5.5 MPa) and porosity percentages (0.57 ± 0.14%) compared to BD (80 ± 17 MPa–1.22 ± 0.31%) and WR (90 ± 22 MPa–1.93 ± 0.12%) (*p* < 0.05). FEA demonstrated that the larger cavity preparation demonstrated higher stress distribution in the root whereas stiffer cement demonstrated lower stress in the root but higher stress in the material. We can conclude that a respected root end preparation associated with cement with good stiffness could offer optimal endodontic microsurgery. Further studies are needed to define the adapted cavity diameter and cement stiffness in order to have optimal mechanical resistance with less stress distribution in the root.

## 1. Introduction

Thanks to the use of magnification devices, micro-instruments, and biocompatible materials, endodontic microsurgery is an optimal option with a success rate of 89–94% when the nonsurgical treatment or retreatment fails to solve the problem [1,2,3,4]. Despite improvements in surgical techniques and materials, the retrograde cavity preparation design and the optimal root-end filling material which insure appropriate mechanical, biological, and physicochemical properties of steel need further investigations.

Different materials were used as root-end filling products such as glass ionomer, plaster of Paris, zinc oxide eugenol, and resin cements which were unable to confront the ideal characteristics of root-end filling materials [5,6,7]. Calcium-silicate-based materials were introduced in the dental market and could be considered the optimal materials for different endodontic treatments [8]. The putty form of these materials is used in several endodontic clinical situations such as pulp cupping, perforations, open apex, apicoectomy, and pulpotomy [4,9,10]. These materials are used in a wide range of endodontic treatments due to their good biological, mechanical, and physicochemical properties [8,9,10,11]. These materials compounds undergo hydrolysis in water to generate greatly soluble calcium hydroxide at the origin of Ca^2+^ ions and alkaline pH [9] which play an important role in the biological reactions and mineralization procedure [12]. Moreover, the mechanical properties of these cements are related to different factors such as the chemical compositions and the hydration process [3,9]. Hou et al. [13] reported that the silica chain and calcium interactions are the main factors of the mechanical strength of these materials.

It was reported that the retrograde cavity design could influence the stress distribution [14]. Until now, there is no study in the literature that has investigated the influence of using calcium silicate materials with different mechanical properties on the distribution of stress concentration. In the present in vitro experiment and finite element analysis (FEA), three calcium-silicate-based cements were mechanically compared. FEA was used previously in dental studies in several dental fields such as coronal restoration [15], root-end surgery [14,16], and implantology [17]. Mineral trioxide aggregate (MTA) and Biodentine™ are the most used calcium-silicate-based materials in putty form. MTA Biorep (Itena Clinical, Paris, France) and Biodentine™ (Septodont, Saint-Maur-des-fossés, France) are powder–liquid calcium-silicate-based cements and in our previous study [3], their biological and physicochemical properties were studied as well as the physicochemical and biological properties of the novel premixed calcium-silicate-based cement (Well-Root™ PT, Vericom, Chuncheon-si, Republic of Korea)). The mechanical properties of the novel premixed product were not found in the literature and the influence of their mechanical properties on the distribution of stress concentration should be investigated. Moreover, the relation between the different calcium-silicate-based cement stiffnesses and the retrograde cavity design should be investigated.

Therefore, the aim of the present study was to investigate the influence of the mechanical properties of three different calcium-silicate-based cements on the stress distribution of three different retrograde cavity preparations. The first null hypothesis was that there is no difference between the compression strength of the different cements and the second one was that the different retrograde cavity preparations could not affect the stress distribution.

## 2. Materials and Methods

### 2.1. Materials and Sample Preparations

Three different calcium-silicate-based cements were used in the study. Biodentine™ “BD” (Powder-liquid cement, Septodont, Saint-Maur-des-fossés, France), MTA Biorep “BR” (Powder-liquid cement, Itena Clinical, Paris, France), and Well-Root™ PT “WR” (Premixed cement, Vericom, Chuncheon-si, Republic of Korea) were prepared following the manufacturer’s instructions [3].

Cylindrical samples (*n* = 10) were prepared using Teflon molds (height: 3.8 mm; diameter: 3 mm) [18]. After filling the molds with the different cements, the samples were put in the dark at 37 °C and 95% humidity for 48 h in a container. After the storage period, the specimens were immersed in distilled water before the mechanical test and porosity investigation at 37 °C for 24 h.

### 2.2. Porosity

After the storage in distilled water for 24 h at 37 °C, the interior texture of BD, BR, and WR were investigated in 3D by using micro-computed X-ray tomography (µCT) (EasyTom 160 from RX Solutions, Chavanod, France). A current of 125 µ and a voltage of 45 kV were used in the execution of imaging procedures using a micro-focused tube supplied with a lanthanum hexaboride (LaB_6_) filament. The source-to-object distance (SOD) and the source-to-detector distance (SDD) were regulated to have a voxel size of around 2.3 µm. The software Xact64 (Version: 22.01.1 2022-03-14, RX Solutions, Chavanod, France) was used to perform the volume reconstruction after the application of ring artifact attenuation and the geometrical corrections. The Avizo software (Version: 3D 2022-2) was used in the image process to remove insignificant small objects, de-noise the images with a median filter, determine the 3D geometrical aspects of the objects of interest, and segment the image intensity in order to reveal the objects of interest [19].

### 2.3. Compression Strength and Modulus

After the storage in distilled water for 24 h at 37 °C, ten specimens from each group were tested through the uni-axial compression test. A universal compression/tensile testing machine (Instron 3345, Norwood, MA, USA) associated with a 1 kN cell force (Class 0.5 following ISO 7500-1) was used at a constant crosshead speed of 0.5 mm/min.

The crosshead displacement in mm and the force in *n* were registered during the test. The stress was calculated in MPa as force dived by the primary section. The strain was acquired by dividing the crosshead displacement by the sample’s primary length. The stress–strain curve was then plotted. The linear section of the stress–strain curve represents the elastic behavior. The compression modulus (Young’s modulus), for each sample is the slope of this linear section that is defined by a linear regression fitting.

The compression strength was calculated in megapascals (MPa) according to the following formula:

σc = 4P/πD^2^

where P is the maximum recorded force during the test and D is the initial sample diameter.

The results of mechanical tests were statistically analyzed using the Kruskal–Wallis test associated with the Tukey test. SigmaPlot release 11.2 (Systat Software, Inc., San Jose, CA, USA) was performed with a statistical significance set at α = 0.05.

### 2.4. Finite Element Analysis (FEA)

An intact human maxillary central incisive, extracted for periodontics problems, was used in the present study. The tooth was scanned by the use of a cone beam computed tomography (CBCT; Vatech, Hwaseong, Republic of Korea) operating with a field of view = 80 × 80 mm^2^ and voxel size = 200 × 200 × 200 mm^3^. The segmentation of the different anatomical structures was based on a previously validated protocol [20]. The segmented 3D image was adjusted to simulate three retrograde conical cavity preparations with an apical diameter of 1 mm (Tip I), 1.4 mm (Tip II), and 1.8 mm (Tip III) after an apical 3 mm resection with 0° bevel angle. The alveolar bone and a periodontal ligament of 0.2 mm around the root were simulated [15]. The segmented 3D image was then meshed using quadratic tetrahedral elements after a convergence test. All dental materials were supposed homogeneous and linearly elastic except the periodontal ligament, which was supposed hyper-elastic. The three root-end filling cements were considered: BD, BR, and WR, and the attributed material properties were referenced from the literature [15] (Table 1). There was a perfect bonding between each component and a vertical load of 150 *n* was applied on the top of the root following published protocols [14,16]. The nodes of the lateral faces of the cortical bone were constrained to prevent displacement. The FEA was conducted on the software Abaqus (Version: Abaqus 6.7 2021, Dassault Systèmes, Vélizy-Villacoublay, France) to calculate the von Mises stresses of the root-end filling.

## 3. Results

### 3.1. Internal Structure (Porosity)

**Figure 1:**
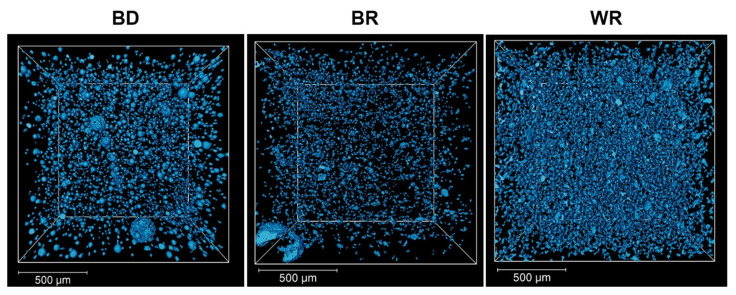
Volume rendering of the segmented pores (blue color) in Biodentine™ (BD), MTA Biorep (BR), and Well-Root PT (WR) obtained by X-ray tomography analysis. The scale bar corresponds to 500 µm in all the images.

All three cements presented pores in their internal structures (Figure 1). BD (1.22 ± 0.31%) and WR (1.93 ± 0.12%) demonstrated slightly higher porosity volume percentages than BR (0.57 ± 0.14%). Therefore, both calcium-silicate-based cements (BD and WR) were characterized by a higher porosity compared to BR (Table 2).

**Figure 2:**
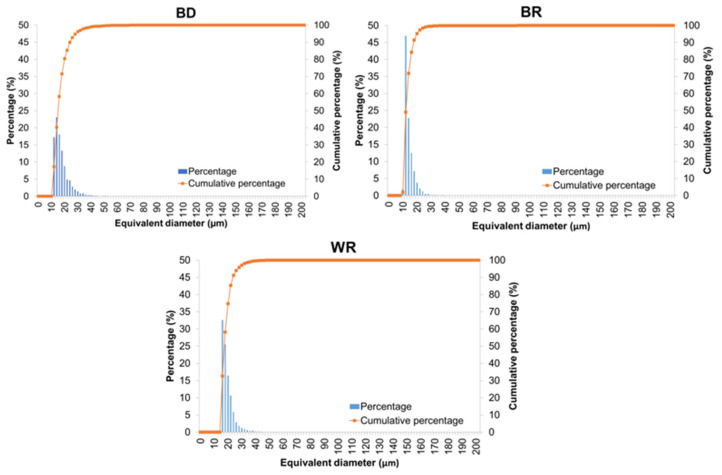
Histograms of the distribution of pore equivalent diameter obtained by X-ray tomography analysis in the case of Biodentine™ (BD), MTA Biorep (BR), and Well-Root PT (WR).

The distribution of equivalent diameters and the average equivalent diameter of the pores were calculated for the three types of samples. The most numerous size range is 14–16 µm in the case of BD, 12–14 µm in the case of BR, and 16–18 µm in the case of WR. For both BD and WR, pores with an equivalent diameter above 30 µm were observed, whereas BR did not exhibit significant pores larger than an equivalent diameter of 30 µm. On average, larger porosity was noted for BD (average equivalent diameter of 16.80 µm) and WR (average equivalent diameter of 18.51 µm) compared to BR (average equivalent diameter of 13.20 µm) (Figure 2).

### 3.2. Compression Strength and Modulus

**Figure 3:**
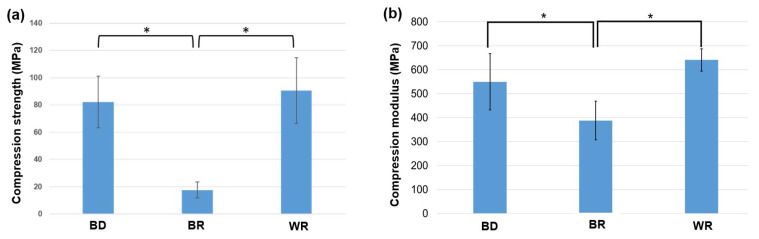
(**a**) Compression strength values (means and standard deviations “MPa”) and (**b**) compression modulus values (means and standard deviations “MPa”) for Biodentine™ (BD), MTA Biorep (BR), and Well-Root PT (WR). (* *p* < 0.05).

The compression strength values for BD (80 ± 17 MPa) and WR (90 ± 22 MPa) were significantly higher than for BR (17.6 ± 5.5 MPa) (*p* < 0.05) after the immersion in water at 37 °C for 24 h (Figure 3a). MTA Biorep presented the lowest compression strength whereas no significant difference was found between WR and BD (*p* > 0.05). For compression modulus, BR cement showed a significantly lower modulus (387 ± 80 MPa) compared to WR (640 ± 46 MPa) and BD (549 ± 117 MPa) after 24 h of immersion in water at 37 °C (*p* < 0.05). No significant difference was found between BD and WR (*p* > 0.05) (Figure 3b).

### 3.3. Finite Element Analysis (FEA)

**Figure 4:**
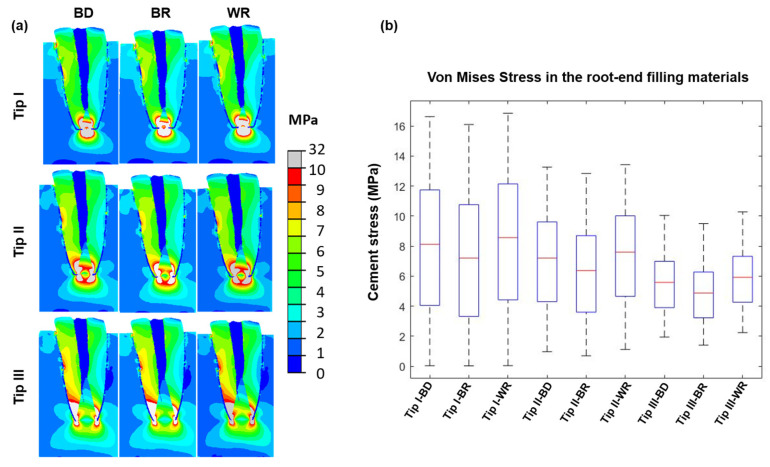
Biomechanical behavior of the resected root with (**a**) stress distribution and (**b**) boxplots presenting the cement stresses for different clinical situations with Biodentine™ (BD), MTA Biorep (BR), and Well-Root PT (WR) and apical diameter of 1 mm (Tip I), 1.4 mm (Tip II), and 1.8 mm (Tip III). On each box, the red central mark indicates the median, and the bottom and top edges of the box indicate the 25th and 75th percentiles, respectively.

Considering all FE models, the highest stress values were located on the apical part of the root and on the root-end filling. For large retrograde cavity preparations, the stress values significantly increase in the root, but significantly decrease in the root-end filling materials. For the same retrograde cavity preparation, the use of a stiffer root-end filling reduces the stress values in the root but increases the stress values in the root-end filling materials. For the smallest retrograde cavity preparation, the stress value varies from 8.56 ± 4.33 MPa to 7.20 ± 4.46 MPa in the root-end filling material. For the largest retrograde cavity preparation, the stress value varies from 5.92 ± 1.93 MPa to 4.87 ± 1.92 MPa in the root-end filling (Figure 4).

## 4. Discussion

As previously described in the literature, bioceramic materials are bioactive products that could offer good biological reactions such as remineralization, antibacterial activity, and antioxidant properties [8,21]. The mechanical properties of endodontic materials could have [11] or no [22] importance due to the location of these materials through the tooth. In addition, the mechanical properties of an endodontic material were considered an unimportant factor in the root canal and as such, they are materials that do not receive high compressive stress [22]. Other studies reported that the mechanical properties of these materials have an important impact on reinforcing the prepared root as well as the resistance against the coronal forces [11,23]. Moreover, the most important factors that could influence the mechanical properties of bioceramic materials are their chemical composition such as the quantity of calcium silicate and the conditions in which these materials should be set up such as the temperature and the humidity [8,9,13,24]. These factors could explain the difference in the mechanical properties of bioceramic materials.

In the present study, BR demonstrated significantly lower compression and modulus strength compared to BD and WR (*p* < 0.05). Therefore, the first null hypothesis must be rejected. These differences could be due to the difference in the chemical composition and the different percentages of calcium silicate in each material [8,9,13]. Moreover, BR demonstrated lower pore percentages compared to BD and WR which could be related to the ability and the speed of hydration of these materials (setting time). Guo et al. [25] reported different setting times of the bioceramic materials and noted that most of the hydration phase occurs during the first several days, despite complete hydration may even take two years. In addition, we can assume that higher porosity percentages and the difference in sizes and pores distributions could affect the compression and modulus strength of calcium silicate materials [8,9]. Moreover, the porosity percentages and pores distribution values were closed in WR and BD which had no significant difference in their compression and modulus strength (*p* > 0.05).

The FEA method was used in the present study to investigate the influence of the mechanical properties of endodontic cements on the stress distribution in endodontic microsurgery. This method presents the advantage of controlling all the conditions and considers various factors that could influence the analysis by using computer software that is inaccessible to test in clinical research [26,27,28]. The applied force (150 *n*) was thought to be sufficient to stimulate the maximum bite force in clinical conditions [14]. All the anatomical considerations that the surgeon could find in a tooth could not be modifiable clinically [14]; therefore, the surgeon could play on the retrograde cavity design and the used bioactive material which could be chosen to ameliorate the quality of the microsurgery treatment and decrease the stress distribution in dentin. In the present study, the material stiffness and retrograde cavity designs affected the stress distribution; thus, the second null hypothesis must be rejected. The larger cavity preparation increased the stress distribution in the root and decreased the stress in the cement material. Therefore, Tip I demonstrated lesser stress in the root and higher stress in the cement material compared to Tip III. In contrast, Kim et al. [14,16] reported that larger retrograde cavity preparations decrease the stress in the root. The difference between these results is that Kim et al. used a higher stiffness for MTA (22 GPa) compared to lower stiffness for the dentin (14 GPa), whereas in our study the cement had lower stiffness values than that used in Kim’s studies [14,16]. Considering that the cement is stiffer than the dentin in their study [14], the FEA will always advantage models presenting larger preparations and thinner dentin walls, which appears to be in contradiction with clinical considerations of preserving more tissue. In parallel, in our study, as the cements had less stiffness than the dentin, the results of FEA concluded that more preparation in dentin generates more stress values in the root. This implies more dentin preservation and again emphasizes the need for adequate retrograde preparation and the influence of this factor compared to other ones such as resection length [29,30]. Moreover, BD and WR generated less stress in the root than BR which had lower stiffness than the other cements (*p* < 0.05), but also a lower compressive strength evaluated experimentally. Therefore, the best choice to decrease the stress distribution in the root is to have a conservative retrograde cavity filled with a cement which presents a higher Young modulus.

Further studies should be performed using more stiffer materials and other retrograde cavity designs applied to different maxillary and mandibular teeth. As a perspective, subsequent research is needed to evaluate the impact of these bioceramic materials in the case of microcracks, a common event in endodontic microsurgery [31]. Moreover, it is important to note that the current study had some limitations that should be taken into account. One of these limitations is that only a single root was simulated, in accordance with previous protocols [14]. However, future studies should also investigate the biomechanical impact of the material on patient-specific models, taking into account the specific anatomy of the root and the dental occlusion, which are known to greatly affect stress distribution [32]. Additionally, the numerical method developed in this study should not lead clinicians to focus solely on the biomechanical aspects of the treatment outcome, as other important factors, such as the use of an operating microscope and ultrasonic instruments, are also crucial for improving the cleaning of the root canal space. Moreover, different immersion periods of calcium silicate materials should be performed to investigate the change of compression and modulus strength in time and their effects on the stress distribution in retrograde treatment. For further clinical applications, future studies should investigate the adapted cavity diameter and cement stiffness in order to have optimal mechanical resistance with less stress distribution in the root.

## 5. Conclusions

Calcium silicate cements have different chemical compositions that play an important role in their biological and mechanical properties. The stiffness of these materials influences the stress distribution in the root and material structure. Moreover, apical preparation design influences stress distribution and the quality of treatment. We can conclude that a respected root-end preparation associated with stiffer cement offer an optimal retrograde treatment with less stress in the root. Therefore, the decrease in stress distribution through the root generates less microfractures; thus, it ameliorates the clinical rate success of the retrograde procedure.

## Figures and Tables

**Table 1:** Material properties used for FEA analysis [15].

Material	Model
Dentine	Linear elastic isotropic E = 14,600 MPa, ν = 0.31
Gutta-Percha	Linear elastic isotropic E = 69 MPa, ν = 0.45
Ligament	Hyper-elastic Ogden order 1; μ = 0.12 MPa, α= 20.9 MPa, D = 10
Trabecular bone	Linear elastic isotropic E = 1300 MPa, ν = 0.3
Cortical bone	Linear elastic isotropic E = 13,000 MPa, ν = 0.3
Biodentine™	Linear elastic isotropic E = 5490 MPa, ν = 0.3
MTA Biorep	Linear elastic isotropic E = 3870 MPa, ν = 0.3
Well-Root PT	Linear elastic isotropic E = 6400 MPa, ν = 0.3

**Table 2:** Pore volume fraction and average pore equivalent diameter obtained by X-ray tomography analysis in the case of Biodentine™ (BD), MTA Biorep (BR), and Well-Root PT (WR).

	BD	BR	WR
Pore volume fraction (%)	1.22 ± 0.31	0.57 ± 0.14	1.93 ± 0.12
Average pore equivalent diameter (µm)	16.80 ± 0.59	13.20 ± 0.09	18.51 ± 0.08

## Data Availability

Not applicable.
